# Identification of the Virulence Factors of *Candidatus* Liberibacter asiaticus via Heterologous Expression in *Nicotiana benthamiana* using *Tobacco Mosaic Virus*

**DOI:** 10.3390/ijms20225575

**Published:** 2019-11-08

**Authors:** Xiaobao Ying, Mengyuan Wan, Linshuang Hu, Jinghua Zhang, Hui Li, Dianqiu Lv

**Affiliations:** 1 Gulf Coast Research and Education Center, University of Florida, Wimauma, FL 33598, USA; yingxb75@ufl.edu; 2 College of Agronomy and Biotechnology, Southwest University, Beibei, Chongqing 400715, China; wmy2018@swu.edu.cn; 3 State Cultivation Base of Crop Stress Biology for Southern Mountainous Land of Southwest University, Beibei, Chongqing 400715, China; 4 Heilongjiang Academy of Agricultural Sciences, Harbin 10086, China; hulinshuang@126.com (L.H.); zhangjinghua010@Hotmail.com (J.Z.)

**Keywords:** Huanglongbing, identify virulence factors, growth inhibition, cell death, subcellular localization, protein–protein interaction, virulence mechanism

## Abstract

Huanglongbing (HLB), also known as citrus greening, is the most destructive disease of citrus worldwide. HLB is associated with the non-culturable bacterium, *Candidatus* Liberibacter asiaticus (*Ca*Las) in the United States. The virulence mechanism of *Ca*Las is largely unknown, partly because of the lack of a mutant library. In this study, *Tobacco mosaic virus* (TMV) and *Nicotiana benthamiana* (*N. benthamiana*) were used for large-scale screening of the virulence factors of *Ca*Las. Agroinfiltration of 60 putative virulence factors in *N. benthamiana* led to the identification of four candidates that caused severe symptoms in *N. benthamiana*, such as growth inhibition and cell death. CLIBASIA_05150 and CLIBASIA_04065C (C-terminal of CLIBASIA_04065) could cause cell death in the infiltrated leaves at five days post infiltration. Two low-molecular-weight candidates, CLIBASIA_00470 and CLIBASIA_04025, could inhibit plant growth. By converting start codon to stop codon or frameshifting, the four genes lost their harmful effects to *N. benthamiana*. It indicated that the four virulence factors functioned at the protein level rather than at the RNA level. The subcellular localization of the four candidates was determined by confocal laser scanning microscope. CLIBASIA_05150 located in the Golgi apparatus; CLIBASIA_04065 located in the mitochondrion; CLIBASIA_00470 and CLIBASIA_04025 distributed in cells as free GFP. The host proteins interacting with the four virulence factors were identified by yeast two-hybrid. The host proteins interacting with CLIBASIA_00470 and CLIBASIA_04025 were overlapping. Based on the phenotypes, the subcellular localization and the host proteins identified by yeast two-hybrid, CLIBASIA_00470 and CLIBASIA_04025, functioned redundantly. The hypothesis of *Ca*Las virulence was proposed. *Ca*Las affects citrus development and suppresses citrus disease resistance, comprehensively, in a complicated manner. Ubiquitin-mediated protein degradation might play a vital role in *Ca*Las virulence. Deep characterization of the interactions between the identified virulence factors and their prey will shed light on HLB. Eventually, it will help in developing HLB-resistant citrus and save the endangered citrus industry worldwide.

## 1. Introduction

Huanglongbing (HLB), also known as citrus greening, is the most destructive disease of citrus. It threatens citrus production worldwide, and all cultivated citrus species are susceptible to it [1,2]. The typical symptoms include asymmetric blotchy molting, stunting, twig dieback, smaller fruits, and uneven fruit color. The infected trees eventually die from HLB [1].

HLB is associated with a fastidious, phloem-restricted bacterium, *Candidatus* Liberibacter spps [3,4]. The Asian form (*Candidatus* Liberibacter asiaticus, *Ca*Las) is transmitted either by Asian citrus psyllid, *Diaphorina citri*, or by grafting. The recent research on HLB focused mainly on either the detection HLB by different methods [5,6,7,8,9,10,11,12,13,14,15,16,17] or citrus responses to HLB at the transcriptional level, translational level, and nutritional-status level [18,19,20,21,22,23,24,25,26,27,28,29]. Efforts were also made to understand the virulence mechanisms of *Ca*Las. For example, a peroxidase that degrades H_2_O_2_ [30], which is a signal in the early response of plant-to-pathogen invasion and in hypersensitive response (HR) [31], has been observed. Similarly, salicylic acid (SA) hydroxylase, which degrades SA [32], was also suggested to play a role in *Ca*Las suppression of plant disease resistance. Recently, a virulence factor, *Ca*Las5315, was identified as the cause of the infiltrated leaves’ death [33]. *Ca*Las5315, also called SDE1 (Sec-delivered effector 1), inhibited the activity of citrus papain-like cysteine proteases, to suppress disease resistance [34]. Although efforts have been made to understand the pathogenesis of *Ca*Las, how the pathogen causes symptoms in citrus is largely unknown. Since *Ca*Las cannot be cultured in vitro, no mutant library is available to identify the virulence factors of *Ca*Las. Similarly, due to shortcomings of transgenic and transient expression of virulence factors, viral vectors can be used efficiently to identify effectors that can affect host development negatively and/or can cause the infected cell death. Compared to the Citrus tristeza virus (CTV), which has a large genome size and is difficult to manipulate, TMV is easy to manipulate; therefore, TMV was used to screen virulence factors of *Ca*Las in *N. benthamiana* in this study.

In this study, four detrimental candidates were identified. CLIBASIA_00470 and CLIBASIA_04025 inhibited plant growth and two other candidates, CLIBASIA_05150 and CLIBASIA_04065C (C-terminal part of CLIBASIA_04065), caused infiltrated leaves’ cell death at five days post infiltration (DPI). The hypothesis of the *Ca*Las virulence was proposed and discussed based on the previous results and our findings. It will help the citrus industry fight HLB.

## 2. Results

### 2.1. Selection of the Putative Virulence Factors

*Ca*Las is an intracellular pathogen that lacks the typical type III secretion system and type IV secretion system [35]. The effectors secrete into host cells mostly via the Sec-dependent pathway. The proteins dependent on the Sec pathway have N-terminal signal peptide (SP) sequences. Therefore, SignalP 3.0 [36], SignalP 4.0 [37], Phobius [38], and TMHMM [39] were used, together, to predict the signal peptide of the selected putative virulence factors. Transmembrane protein topology prediction and signal peptide prediction were highly similar due to hydrophobic regions present in both the transmembrane domain and signal peptide. Therefore, Phobius was used to distinguish the signal peptide and transmembrane protein [38]. The pathogen also has type I secretion system (TISS) to secret protein into host cells; therefore, hemolysin and serralysin, which are dependent on TISS, were also included in the list (Table 1).

### 2.2. Agroinfiltration of Putative Virulence Factors in N. benthamiana

**Figure 1:**
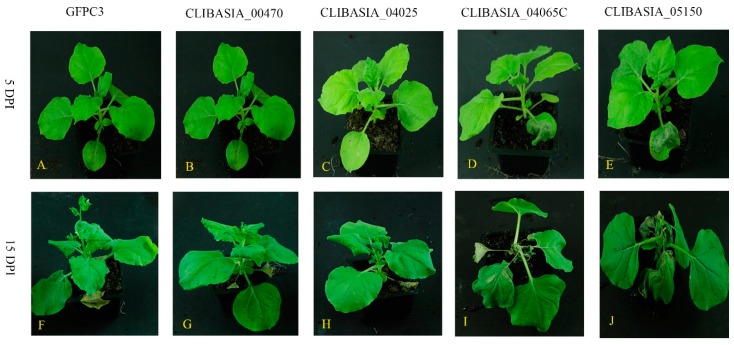
*N. benthamiana* expression four putative virulence factors showed strong phenotypes. By expression the total 60 selected putative virulence factors via TMV in *N. benthamiana*, four putative virulence factors showed strong phenotypes in *N. benthamiana*. The photos in the upper panel were taken at 5 days post infiltration (DPI). (**A**) TMV-GFPC3 was used as control, no cell death occurred in the infiltrated leaves; (**B**) the leaves infiltrated with CLIBASIA_00470 were similar to control; (**C**) leaves infiltrated with CLIBASIA_04025 resembled control TMV-GFPC3; (**D**) CLIBASIA_04065C caused the cell death in the infiltrated leaves; (**E**) expression CLIBASIA_05150 resulted in cell death in the infiltrated leaves. The photos in lower panel were taken at 15 DPI. Plants expressed CLIBASIA_00470 (**G**) and CLIBASIA_04025 (**H**) showed a stunting phonotype compared with TMV-GFPC3 (**F**); CLIBASIA_04065C (**I**) and CLIBASIA_05150 (**J**) caused severe symptoms in systemic leaves, including cell death.

The agroinfiltration of *N. benthamiana* was first tested by using pBI121-TMV-GFPC3 vector, which has the green fluorescent protein (GFP). The GFP signal was observed under UV light at 4 days post infiltration (DPI) in the infiltrated leaves, and virus movement was detected in systemic leaves at 10 DPI (Supplementary Figure S1). Infiltration of the 60 putative virulence factors led to several phenotypes in *N. benthamiana* which were classified into four groups (Table 1). Group 1 showed strong phenotypes. CLIBASIA_05150 caused the infiltrated leaves’ death at 5 DPI (days post infiltration) (Figure 1E). CLIBASIA_04065, which is above the package limitation of TMV, has ATP binding activity by prediction with PSIPRED server [40]. If we divided the protein into two parts evenly, the C-terminal part (CLIBASIA_04065C) could cause infiltrated leaves’ cell death at 5 DPI (Figure 1D). After two weeks of infiltration, the plants expressed CLIBASIA_00470, and CLIBASIA_04025 showed strong symptom of stunting (Figure 1G, H). The height of *N. benthamiana* expression CLIBASIA_00470 and CLIBASIA_04025 was about 66% of the height of plant expression TMV-GFPC3 at 15 DPI (Supplementary Table S2). However, the *N. benthamiana* leaves infiltrated with CLIBASIA_00470 and CLIBASIA_04025 were similar to those infiltrated with TMV-GFPC3 at 5 DPI (Figure 1A–C). Cell death occurred in the systemic leaves of *N. benthamiana* expressing CLIBASIA_04065C and CLIBASIA_05150 at 15 DPI (Figure 1I, J). Forty-two putative virulence factors belonged to group 2, which showed similar symptoms to the TMV-GFPC3 control. Group 3 had two candidates, CLIBASIA_04040 and CLIBASIA_05640, that attenuated the TMV symptoms. The last group of candidates did not show any symptom in systematic leaves. The common feature of group 4 is the large DNA size that is beyond TMV package limitation; therefore, TMV cannot move to upper systemic leaves.

### 2.3. The Four Virulence Factors Functioning at the Protein Level

**Figure 2:**
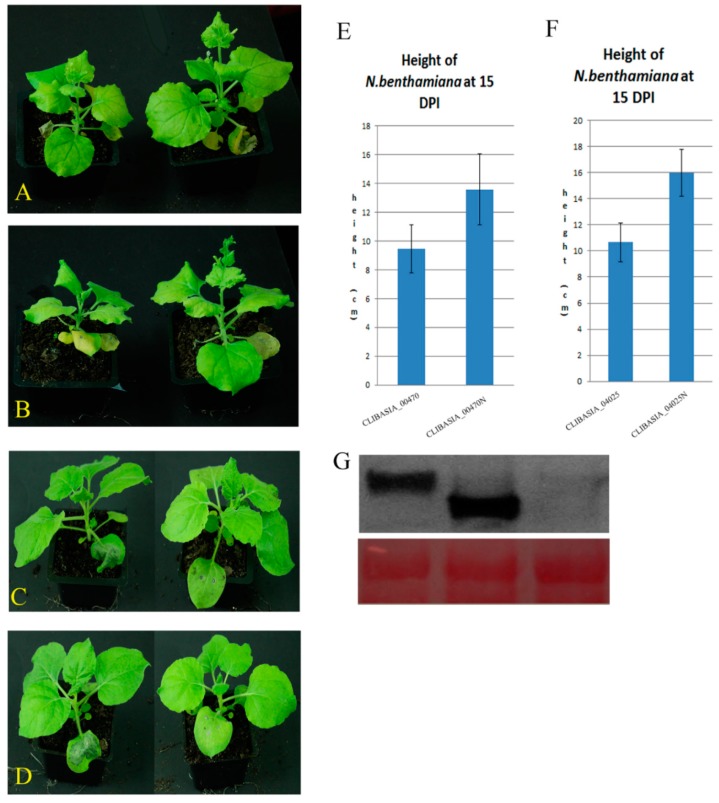
The four virulence factors functioned at protein level. (**A**) *N. benthamiana* infiltrated with mutated CLIBASIA_00470 (CLIBASIA_00470 N, converted start codon ATG to stop codon TAA on right) showed normal growth compared with *N. benthamiana* infiltrated with CLIBASIA_00470 (on left, stunting); (**B**) Showed the null mutation of CLIBASIA_04025 (right) lost the function of growth inhibition compared with CLIBASIA_04025 (left). (**C**) Left was HA- CLIBASIA_05150, it had the same function as native protein, caused the infiltrated leaves cell death at 5 DPI; right was the null mutation, no cell death observed in the infiltrated leaves at 5 DPI. (**D**) Left infiltrated with CLIBASIA_04065C-HA, the cell death was observed in the infiltrated leaves at 5 DPI as wild type of CLIBASIA_04065C; right was frame shifting mutation, no cell death in the infiltrated leaves. (**E**) The different height of *N. benthamiana* infiltrated with CLIBASIA_00470 or CLIBASIA_00470N at 15 DPI. (**F**) The different height of *N. benthamiana* infiltrated with CLIBASIA_04025 or CLIBASIA_04025N at 15 DPI. (**G**) Western blotting detected HA-fused protein at 5 DPI. Left: infiltrated with HA-GFPC3 (28 kDa), middle infiltrated with HA-CLIBASIA_05150 (22.23 kDa), right infiltrated with CLIBASIA_04065C-HA (23.56 kDa). Lower panel was loading control.

To determine the four detrimental virulence factors functioning at the protein level instead of the RNA level, the mutations were introduced into the four effectors, to eliminate the functional proteins. Start codon ATG was converted into stop codon TGA for *CLIBASIA_00470*, *CLIBASIA_04025*, and *CLIBASIA_05150*. By mutation, the virulence factors could not translate into proteins. For *CLIBASIA_04065C*, the frameshifting was employed because an in-frame start codon ATG was inside of the DNA fragment. The mutated CLIBASIA_00470, CLIBASIA_04025, CLIBASIA_05150, and CLIBASIA_04065C could not inhibit plant growth nor cause cell death (Figure 2A–D). The height of *N. benthamiana* infiltrated the mutated CLIBASIA_00470N, and CLIBASIA_04025N was similar in height to the TMV-GFPC3-infiltrated plants (Supplementary Table S2), the height was 50% more than the plant expression in the wild type *CLIBASIA_00470*, *CLIBASIA_04025*, at 15 DPI (Figure 2E,F). No cell death was observed in the *N. benthamiana* infiltrated with CLIBASIA_05150N (Figure 2C). Frame-shifted *CLIBASIA_04065C* could not cause infiltrated leaves; death (Figure 2D). To confirm the phenotypes were caused by protein, HA-tag was fused at the N-terminal of *CLIBASIA_05150*, and HA-tag was added at the C-terminal of *CLIBASIA_04065C*. The HA fused proteins showed the same phenotypes as the wild type proteins (Figure 2C–D). The proper size of fusion proteins HA-GFP, HA-CLIBASIA_05150, and CLIBASIA_04065C-HA were detected by Western blot (Figure 2G).

### 2.4. The C-Terminal of CLIBASIA_05150 Was Critical in Causing Cell Death in the Infiltrated Leaves

**Figure 3:**
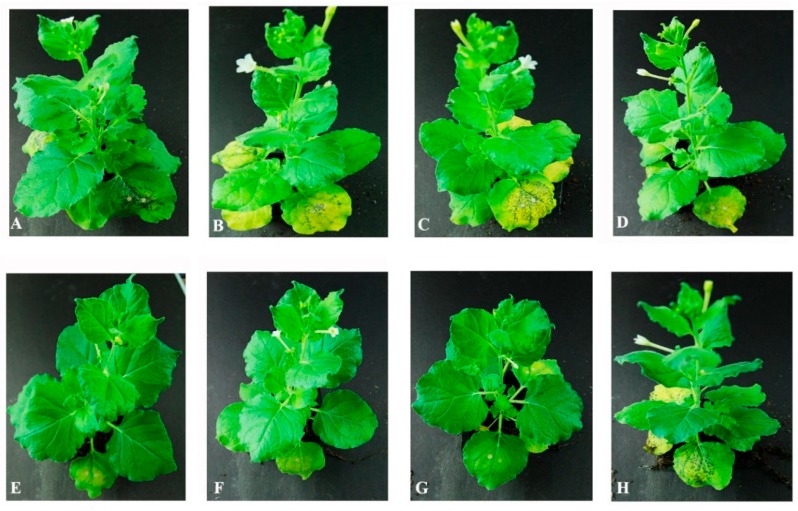
Determination of the functional domain of CLIBASIA_05150. To determine the functional domain of CLIBASIA_05150, deletion assay was performed. Pictures were taken at 6 DPI. (**A**) The full length of CLIBASIA_05150 (cell death); (**B**) deleted 1/3 from N-terminal (cell death); (**C**) deleted 1/3 from middle (cell death); (**D**) deleted 1/3 from C-terminal (no cell death); (**E**) only included the C-terminal (no cell death); (**F**) it included N-terminal, middle part and 1/4 of C-terminal (no cell death); (**G**) it included N-terminal, middle part and 1/2 of C-terminal (no cell death); (**H**) it included N-terminal, middle part and 3/4 of C-terminal (cell death). The black spots in the infiltrated leaves indicated the cell death.

To determine the functional domain of CLIBASIA_05150, series deletions were performed. First, the full-length protein was divided into three parts evenly to get three deletions: (1) deleted the N-terminal part, (2) deleted the middle part, and (3) deleted the C-terminal part. Infiltration N. benthamiana with the three constructs, respectively, only the constructs without the C-terminal part could not cause infiltrated-leaf cell death. To narrow down the functional domain of C-terminal part, the C-terminal part was divided into four parts evenly. The 3/4 length of C-terminal showed the identical function as the full-length C-terminal part (Figure 3). 

### 2.5. The Subcellular Localization of the Four Putative Virulence Factors

**Figure 4:**
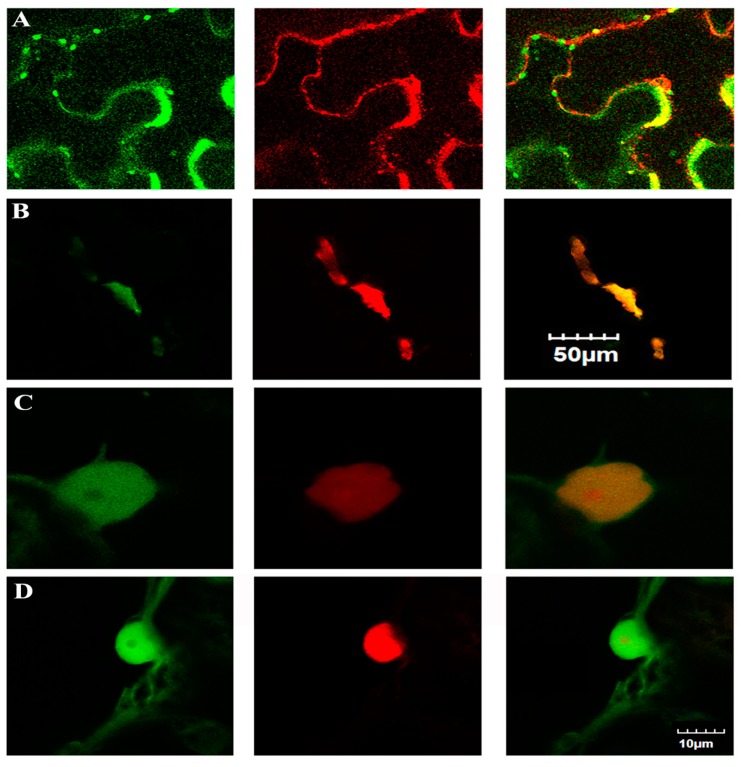
Subcellular localization of CLIBASIA_05150, CLIBASIA_04065, CLIBASIA_00470, and CLIBASIA_04025. (**A**) Showed the subcellular location of CLIBASIA_05150. The left one showed CLIBASIA_05150-GFP fusion protein, middle showed RFP targeted to Golgi apparatus, the right one showed overlay of left and middle. (**B**) Showed the subcellular localization of CLIBASIA_04065. Left showed the CLIBASIA_04065-GFP fusion protein, middle was RFP targeted to mitochondria, right showed overlay of left and middle. (**C**,**D**) Showed the subcellular localization of CLIBASIA_00470 and CLIBASIA_04025, respectively. Left showed GFP fusion protein, middle showed RFP with nuclear localization signal (NLS), right showed overlay of left and middle. **A** and **B** were the same scale; **C** and **D** were the same scale.

The subcellular localization of the four putative virulence factors was determined via transient expression effector-GFP fusion protein in *N. benthamiana* and observed under a laser scanning microscope. Post infiltration 48 h, the leaves were observed under an Olympus FV1000 MPE multiphoton laser scanning microscope. The distribution patterns of the CLIBASIA_05150-GFP and CLIBASIA_04065-GFP fusion proteins in *N. benthamiana* cells were different from the control, free GFP. The untagged GFP distributed in the cytosol and to the nuclear lumen, but CLIBASIA_05150-GFP fusion protein was small green dots under the confocal microscope. CLIBASIA_04065-GFP showed many green dots, green cycles, or grain-like shape (Supplementary Figure S2). To locate the specific location of the two fusion proteins, the red fluorescent protein (RPF)-targeted [41] to different organelle was co-infiltrated with the two GFP-fusion proteins, respectively. CLIBASIA_05150-GFP was overlapping with RFP-Golgi, and CLIBASIA_04065-GFP was overlapping with RFP-Mitochondrion (Figure 4). The distribution patterns of CLIBASIA_00470-GFP and CLIBASIA_04025-GFP were similar to free GFP (Supplementary Figure S2). RFP with nuclear localization signal (NLS) [42] was co-infiltrated with the two fusion proteins to determine whether they located in nuclei. The RFP-NLS was merged with CLIBASIA_00470-GFP or CLIBASIA_04025-GFP fusion proteins, and the two fusion proteins did not overlap with RFP-NLS (Figure 4). 

### 2.6. Identification of the Host Proteins Interacting with the Virulence Factors

The autoactivations of CLIBASIA_00470 and CLIBASIA_04025 were eliminated by deletion 11 aa at N-terminal of the mature CLIBASIA_00470 and 25 aa at the C-terminal of CLIBASIA_04025. yeast two-hybrid (Y2H) was performed to identify the host proteins interacting with virulence factors. Seventeen genes (41 isolates) were identified to interact with CLIBASIA_00470. Ten genes (13 isolates) interacted with CLIBASIA_04025. The prey of CLIBASIA_05150 belonged to 6 genes (8 isolates). Only one prey was identified to interact with CLIBASIA_04065 (Table 2).

Among the prey of CLIBASIA_00470, three isolates were polyubiquitin-like proteins, which are the precursors of free ubiquitin [43]. Galactinol-sucrose galactosyltransferase 2-like protein that are involved in raffinose metabolism took the first place among the prey of CLIBASIA_00470 (13/41). Raffinose is the major soluble carbohydrate in roots. DnaJ like protein, a molecular chaperone of Hsp70, took the second place (7/41). Six isolates were agglutinin-2-like protein, an l-type lectin. The identical homolog of XM_006495169, agglutinin-2-like protein, in Volkamer lemon was induced by SA treatment or water deficit [44]. Two isolates were NDR1/HIN1-like protein 13, a protein confers resistance to bacterial pathogen [45]. One prey was thaumatin-like protein 1b, also known as pathogenesis-related protein 5b (PR5b), that has antifungal activity [46]. XM_006470373 encodes an l-ascorbate oxidase-like protein. Ascorbate oxidase affects plant development and interaction with microbes by regulation of the overall redox state [47]. XM_006473640 annotated as cinnamyl alcohol dehydrogenase 6 that catalyzes the final step specific for the production of lignin monomers and XM_006466284 annotated as 8-hydroxygeraniol dehydrogenase-like that is involved in the biosynthesis of oxogeranial from hydroxygeraniol, a precursor of the terpenoid indole alkaloids. XM_025093156 encoded a heat-shock 70 kDa protein 6. Hsp70 proteins function as the central hub of cellular network of molecular chaperones and folding catalysts. Hsp70 proteins assist the de novo folding nascent proteins and restore the conformation of non-native proteins under stress [48,49]. XM_006486253 encoded a phosphoinositide phospholipase C 2-like protein. Phosphoinositide-specific phospholipase C (PI-PLC) enzymes function immediately downstream of FLAGELLIN SENSING2 (FLS2) and are crucial for plant defense response [50]. A TPR-DOMAIN SUPPRESSOR OF STIMPY (TSS) protein was encoded by XM_025092931. UPF0587 protein C1orf123 also interacted with CLIBASIA_00470. Two proteins, XM_006469523 and XM_006479243, were uncharacterized proteins. However, the ortholog of XM_006479243 from *Arabidopsis* annotates as a putative ubiquitin ligase.

**Figure 5:**
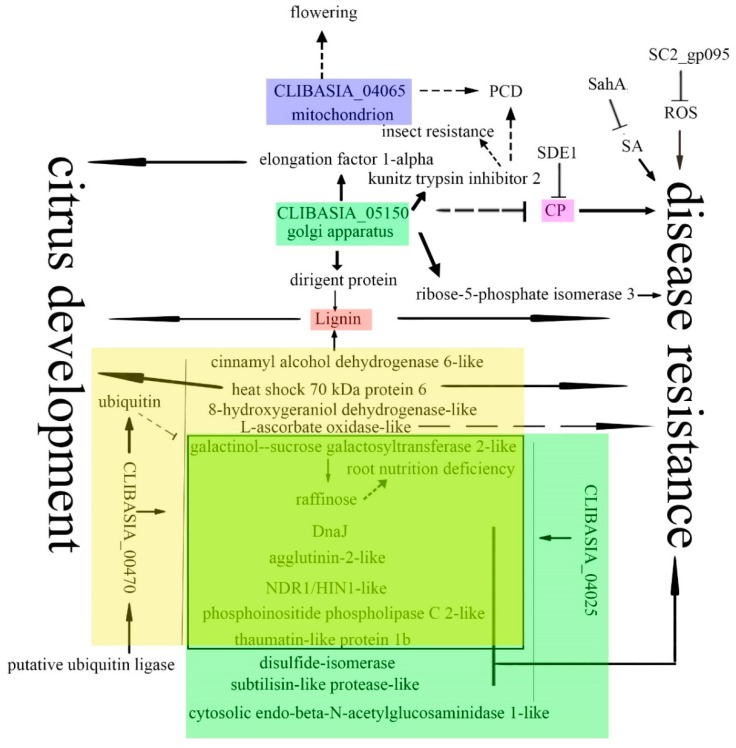
The hypothesis of the virulence mechanisms of *Ca*Las. It was reported previously that *Ca*Las inhibited citrus disease resistance by degradation defense signals SA (salicylic acid) and H_2_O_2_ and inhibition CPs (cysteine protease). CLBASIA_05150 located in Golgi apparatus. It interacted with proteins involved in disease resistance, plant development, and protein synthesis. CLBASIA_05150 may interfere with the transportation of prey to the destination. CLBASIA_04025 and CLBASIA_00470 had the overlapping profiles of prey (indicated by the rectangular box). The identified prey included proteins involved in disease resistance, as well as plant development. CLBASIA_00470 may downregulate targets by ubiquitin-mediated protein degradation. CLIBASIA_04065 located in mitochondria. The prey may play a role in off-season flowering and programmed cell death (PCD). The solid line indicated the confirmed interaction or the known function of the protein. The dashed line indicated the speculation of interaction between proteins or the unknown function of proteins. The ‘T’ shape line indicated the inhibition function.

CLIBASIA_04025 caused the same phenotype in *N. benthamiana* as CLIBASIA_00470. It was not surprising that the prey of CLIBASIA_04025 overlapping with the prey of CLIBASIA_00470. XM_006495169, XM_006489869, XM_006486253, and XM_006473608 interacted with the two virulence factors (Figure 5). XM_006485422 was annotated as a DnaJ protein homolog. XM_006489870 annotated as XM_006489869, galactinol-sucrose galactosyltransferase. XM_006465840 annotated as subtilisin-like protease-like protein. Subtilisin-like proteases belong to serine peptidases, mostly present in plants. Subtilisin-like proteases contribute to plant defense to pathogen not only by mounting an effective defense response but also by causing direct damage to the pathogen [51]. XM_006486194 was annotated as cytosolic endo-beta-N-acetylglucosaminidase 1-like protein. The endo-beta-N-acetylglucosaminidase genes controlled free N-glycan levels in *Arabidopsis* [52]. The N-glycan was associated with fruit ripening [53]. XM_006472735 annotation was disulfide-isomerase. Disulfide-isomerase (DI) is an enzyme that catalyzes disulfide formation and isomerization and function as a chaperone to inhibit aggregation [54]. DI has thiol redox activity. In response to pathogen infection, DI plays two roles: (1) the internalization of some pathogens and (2) the promotion of increased ROS in cells [55].

Three isolates of XM_006474664 interaction with CLIBASIA_05150 were cysteine protease (CP), which interacted with another effector of *Ca*Las, SDE1 [34]. The rest of the isolates, XM_006471352, XM_025096339, XM_006441377, XM_006492080, and XM_006485840, were annotated as uncharacterized protein, ribose-5-phosphate isomerase 3, Kunitz trypsin inhibitor 2, dirigent protein 16, and elongation factor 1-alpha, respectively. Ribose-5-phosphate isomerase 3 (RPI3) catalyzes the reversible conversion of ribose-5-phosphate to ribulose 5-phosphate and plays a role in defense response to bacterium [56]. Kunitz trypsin inhibitor 1 from *Arabidopsis* antagonizes the cell death triggered by phytopathogens [57]. Kunitz trypsin inhibitor also functions in plant defense against insect pests [58]. Dirigent proteins are the regulator of plant growth and development, as well as a plant modulator in cell-wall metabolism during the abiotic and biotic stress function via regulation lignin and lignan biosynthesis [59]. Elongation factor 1-alpha promotes the GTP-dependent binding of aminoacyl-tRNA to the A-site of ribosomes during protein biosynthesis. It may be the rate-limiting factor for protein synthesis [60].

Only one prey interacted with CLIBASIA_04065, which was orange1.1g037576m. The *Arabidopsis* ortholog of orange1.1g037576m locates in the mitochondrion and affects flowering.

## 3. Discussion

Because HLB is a major threat to the citrus industry [1], numerous research studies have been conducted, including studies on the identification of the causal agent [3,61], the cellular structure change of the infected trees [27,62], confirmation of the causal agent from putative-infected trees [5,9,10,12,63], and detection of the putative-infected trees [6,8,13,14]. In spite of the studies that were done to understand the pathogenesis of *Ca*Las [30,32,33,34,64] and the gene regulation of *Ca*Las [65], the virulence mechanism is still largely unknown, because of the unavailability of the mutant library. The accessibility of genomic information of *Candidatus* Liberibacter [4,35,66,67] makes screening the putative virulence factors possible by expression candidates in planta. CRISPR is a powerful tool to generate canker-resistant citrus [68,69]. However, it is unable to apply CRISPR to citrus to generate HLB resistance citrus without a deep knowledge of the mechanisms of HLB development [70]. Our research on screening the virulence factors by using TMV and *N. benthamiana* aimed at gaining more insight into the pathogenesis of *Ca*Las.

*Ca*Las belongs to intracellular bacteria. The pathogen lacks a type-three secretion system [35]; therefore, the putative virulence factors mainly depend on the Sec-dependent machinery to enter host cells. A virulence factor, tengu-su inducer (TENGU) from onion yellows phytoplasma, was identified by bioinformatics prediction, followed by identification of the putative virulence factors by expression in *N. benthamiana* via viral vector, and confirmed with transformation *Arabidopsis* [71]. The virulence factor caused plant sterility by downregulating of the jasmonic acid and auxin pathways [72]. Because *Ca*Las cannot be cultured in vitro, expression of the putative virulence factors in plant was needed to identify the virulence factors. A time- and labor-saving system was needed for large-scale screening of the putative virulence factors of *Ca*Las. *N. benthamiana* was used to identify the virulence factor of *Ca*Las that induced cell death by transient expression [33]. However, transient expression cannot identify the virulence factors that inhibit plant development. Viral vector can be used to identify the effectors that regulate plant development negatively, besides the lethal effectors. Though CTV could express GFP by agroinfiltration in *N. benthamiana* and inoculated citrus by stem-slash inoculation [73], the large genome size of CTV and the long amount of time needed for CTV replication in *N. benthamiana* and observation phenotype in citrus make CTV undesirable for large-scale screening putative virulence factors of *Ca*Las. *Ca*Las can multiply in tobacco (*N*. *tabacum* cv. Xanthi) and causes symptoms [74]; thus, it is possible that the virulence factors target the conserved pathway in tobacco as in citrus. Hence, *N. benthamiana*, a model plant for plant–pathogen interaction [75], is used to screen putative virulence factors of *Ca*Las at a large scale, via agroinfiltration, to express effectors by TMV.

Sixty putative virulence factors were screened (Table 1). The selected 60 candidates were expressed in *N. benthamiana* by TMV via agroinfiltration. Based on the phenotypes of *N. benthamiana* expression the selected candidates, the 60 candidates were classified into four groups: (1) four candidates showed severe symptoms, in the form of growth inhibition or cell death; (2) plants expression 42 candidates were similar to the plant infiltrated with TMV-GFPC3; (3) two candidates attenuated the TMV symptom; (4) twelve candidates did not show TMV symptoms in the systemic leaves. The two low-molecular-weight proteins, CLIBASIA_00470 and CLIBASIA_04025, showed similar symptoms. *N. benthamiana* expressing the two candidates showed a stunting phenotype compared with TMV-GFPC3 control at 15 DPI (Figure 1 and Figure 2). It is the first report that virulence factors of *Ca*Las inhibited plant growth. One of the symptoms for HLB-infected citrus is stunting. Ubiquitin-proteasome regulates plant development by degradation proteins [76] and functions as a signal pathway in plant immune response [77]. Pathogens encode virulence factors to disturb the plant host’s ubiquitin system as a virulence mechanism [78,79]. CLIBASIA_00470 interacted with polyubiquitin and other proteins. The ortholog of XM_006479243 in *Arabidopsis* (AT3G07060.1) is annotated as a ubiquitin ligase. Therefore, CLIBASIA_00470 may use the ubiquitin system to downregulate the host proteins and may subject itself to the regulation of the host ubiquitin system (Figure 5). CLIBASIA_00470 and CLIBASIA_04025 share major similarities: (1) the *N. benthamiana* expressed the two effectors showing the same stunting phenotype; (2) the two proteins both located in the cytoplasm; (3) the profiles of host proteins interacting with the two virulence factors were overlapping largely. Therefore, the two proteins may function redundantly. Because only part of the two virulence factors was used in Y2H, the identified prey of the two effectors cannot represent all the host proteins interacting with them. How the two effectors function in HLB development needs to be determined further.

Mitochondria play an important role in plant defense upon pathogen infection by integration and amplification of diverse signals, such as salicylic acid, nitric oxide, reactive oxygen species (ROS), or pathogen elicitors at early stages and promotion-programmed cell death (PCD) at advantage stages [80]. On the other hand, pathogens target mitochondria to cause disease development. For example, HopG1 from *P. syringae* pv. tomato DC3000 is a virulence factor located in mitochondria. The HopG1 transgenic *Arabidopsis* and *Nicotiana tabacum* showed a stunting phenotype. Constitutive expression of HopG1 in transgenic planta resulted in reduced respiration rates and an increased basal level of reactive oxygen species. These findings suggest that HopG1 promotes disease by disrupting mitochondria functions with specific subcellular localization [81]. The sieve element is a highly specific cell for transportation by losing important organelles, such as the nucleus, the vacuole, ribosomes, and Golgi apparatus. However, smooth endoplasmic reticulum (ER), mitochondria, sieve element plastids, and phloem proteins remain in sieve element [82]. CLIBASIA_04065 located in mitochondria (Figure 4B) and the plants expressing CLIBASIA_04065C via TMV caused the infiltrated leaves death (Figure 1D). TMV can express heterogeneous protein at a high level up to 10 percent of total soluble protein in plants [83]. The severe symptom of infiltrated leaves death may relate to the large amount of CLIBASIA_04065C. Only one gene, *orange1.1g037576m*, from citrus was found to interact with CLIBASIA_04065. The *Arabidopsis* ortholog of *orange1.1g037576m* is AT3G61930.1. The annotated localization is mitochondrion and expressing at the flowering stage. It is possible that the *Ca*Las replicates to a threshold that CLIBASIA_04065 disrupts the structure and function of mitochondria, contributing to the death of the HLB-infected citrus. However, it may affect citrus flowering when at a lower concentration.

The plant Golgi apparatus is the factory of polysaccharides and glycoproteins, and it involved the transportation of proteins to their destinations [84,85]. Moreover, the Golgi apparatus plays an important role in disease resistance. For instance, Golgi phosphate transporter PHT4; 6 affected plant development and disease resistance [86]. SYP4 [87], Arabidopsis ceramidase (AtACER) [88], and CMPG1-V [89] are in the Golgi apparatus and affect plant-disease resistance. CLIBASIA_05150 located in Golgi (Figure 4B) showed the same symptom to CLIBASIA_04065C (Figure 1E). CLIBASIA_05150 interacted with three genes, XM_006474664 (NM_001288897), XM_025096339, and XM_006492080, which were related to plant-disease resistance. XM_006474664, a cysteine protease (CP), was reported to interact with SDE1, which was a conserved protein in all *Ca*Las isolates. SDE1 suppresses citrus defense responses by inhibiting CP activities [34]. Interestingly, CLIBASIA_05150 interacted with XM_006474664 as well; therefore, XM_006474664 may play an important role in citrus resistance to HLB. Dirigent proteins are involved in lignin and lignan production [90]. Dirigent proteins participate in plant response to abiotic stress and biotic stress [59]. For instance, overexpression of a soybean dirigent (DIR) gene *GmDIR22* in the susceptible soybean cultivar ‘Dongnong 50′ improved its resistance to *Phytophthora sojae* [91]. XM_025096339 encodes a ribose-5-phosphate isomerase 3 which is involved in the response of *Arabidopsis* to bacterium [56]. CLIBASIA_05150 is located in the Golgi apparatus and interacts with important proteins involved in development and disease resistance. CLIBASIA_05150 may lead to retention of the host proteins, and this contributes to HLB development.

The tolerance of citrus to *Ca*Las relies on basal resistance [20]. Based on our data, *Ca*Las may target different pathways for the virulence. Lignin may be an important target of *Ca*Las. Cell-wall swell is an early sign of HLB infection [62]. Lignin is the main component of the cell wall. XM_006473640 and XM_006492080 involved in lignin synthesis interacted with two identified virulence factors. Less content of lignin in cell walls may facilitate the bacteria to move between cells, and also may contribute to cell-wall swell. Moreover, less lignin in cell walls may affect transportation and plant development. Phloem regeneration contributed to the tolerance of citrus to HLB [92]. Phloem is the battlefield of citrus and *Ca*Las. The biggest category of proteins interacting with virulence factors was related to plant response to bacterial infection. Agglutinin-2-like protein, NDR1/HIN1-like protein 13, phosphoinositide phospholipase C 2-like protein, subtilisin-like protease-like protein, disulfide-isomerase, cysteine protease, and ribose-5-phosphate isomerase 3 are involved in disease resistance. CP is the hub of plant immunity, and the pathogen effectors target CP [93]. SC2_gp095, SahA, and SDE1 suppressed citrus resistance to HLB by the degradation of H_2_O_2_ [30] and salicylic acid [32], and by inhibiting activity of CP [34] (Figure 5). Suppression of citrus-disease resistance is an important part of *Ca*Las virulence. Kunitz trypsin inhibitor is involved in PCD and resistance to insects. The identified Kunitz trypsin inhibitor 2 that interacted with CLIBASIA_05150 may contribute to the virulence of *Ca*Las by the suppression of PCD or making the infected citrus favorable to psyllid.

Raffinose’s metabolism was drastically altered upon HLB infection [18]. CLIBASIA_00470 and CLIBASIA_04025 interacted with XM_006489869 and XM_006489870, the enzymes that catalyze the chemical reaction: alpha-d-galactosyl-(1→3)-1d-myo-inositol + sucrose ⇋ myo-inositol + raffinose. It is the first evidence of *Ca*Las effectors directly related to raffinose metabolism. Since raffinose is the major soluble carbohydrate in the root, the altered raffinose metabolism may contribute to the root decline in HLB-infected citrus. The protein metabolism was altered in HLB-infected citrus. We showed that the elongation factor 1-alpha, which participates in translation, interacting with CLIBASIA_05150. Two molecular chaperones (DnaJ/Hsp70) were found to interact with virulence factors. DnaJ/Hsp genes were highly depressed in HLB-infected mandarins [94], and Hsp70 was downregulated in HLB-infected grapefruit [29]. In spite of the long-time finding of downregulation of DnaJ and Hsp70 in HLB-infected citrus, it is the first time the virulence factors of *Ca*Las interacting with the two molecular chaperones was shown. It is possible that CLIBASIA_00470 and CLIBASIA_04025 downregulated the host proteins via ubiquitin-mediated protein degradation. Downregulation of molecular chaperone amplifies the virulence. Therefore, ubiquitin-mediated protein degradation must play a critical role in HLB symptoms’ development. Based on the previous reports and our results, a hypothesis of *Ca*Las virulence is proposed (Figure 5). The effectors of *Ca*Las target to different plant organelles to disturb the normal functions of cells. It was reported that *Ca*Las inhibited citrus disease resistance. We propose that *Ca*Las inhibits citrus disease resistance and affects citrus development intricately. The important targets of *Ca*Las were regulated by different effectors. Ubiquitin-mediated protein degradation system may play a pivotal role in the downregulation of host proteins. CLIBASIA_00470 interacted with ubiquitin, as well as other host proteins, including a putative ubiquitin ligase. It is plausible that CLIBASIA_00470 downregulates other host proteins by ubiquitin-mediated protein degradation. CLIBASIA_00470 may downregulate genes that relate to lignin synthesis, raffinose metabolism, and defense response to bacterium and fungus. The downregulation of molecular chaperone 70 and its chaperone Hsp40/DnaJ might amplify the detrimental effects of *Ca*Las infection, by making other host proteins unstable. Lignin contributes to plant development and response to abiotic and biotic stresses [95]. Raffinose may relate to the root decline in the HLB-infected citrus since raffinose is the major soluble carbohydrate in the root. CLIBASIA_00470, CLIBASIA_04025 and CLIBASIA_05150 interacted with multiple defense-related proteins. Among them, CP interacted with two virulence factors, SDE1 and CLIBASIA_05150. XM_006486194 interacted with CLIBASIA_04025 and may affect the citrus fruit ripening. CLIBASIA_05150 localized in Golgi. The specific subcellular localization may lead to retention of the interacting host proteins in Golgi; therefore, it disturbs the normal function of the interacting proteins. The only prey of CLIBASIA_04065 annotated as a hypothetical protein, but its ortholog in *Arabidopsis* affects flowering. The relationship between off-season flowering in HLB-infected citrus and *orange1.1g037576m* needs to be determined. Two proteins involved in cell death, Hsp70 and Kunitz trypsin inhibitor, were found to interact with virulence factors CLIBASIA_00470 and CLIBASIA_05150. It is plausible that *Ca*Las inhibits the host defense responses and cell death for its own fitness. Characterization of the functions of the identified citrus proteins which interacted with the four virulence factors will shed light on the mechanisms of disease development. In the long run, it will help in developing HLB-resistant citrus to save the endangered citrus industry.

## 4. Materials and Methods

### 4.1. Plant Material and Growth Conditions

*N. benthamiana* plants were grown under the controlled condition of 16 h light/8 h dark, at 25 °C.

### 4.2. Prediction of Signal Peptides of Putative Virulence Factors

The signal peptide of putative virulence factors was predicted by combination of SignalP 3.0 [36], SignalP 4.0 [37], Phobius [38], and TMHMM [39]. The sequences were input manually.

### 4.3. Construction of Plasmids

#### 4.3.1. Construction of Plasmids for Screening Putative Virulence Factors of *Ca*Las

TMV was used to express the putative virulence factors of *Ca*Las in *N*. *benthamiana*. The vectors used in this study were based on pBI121 [96]. The putative virulence factors without the signal peptide were inserted between *Pac*I and *Xho*I restriction enzyme sites of p30B-GFPC3 to construct an intermediate vector [83]. The 5′ UTR of TMV following cauliflower mosaic virus promoter (CaMV 35S) was amplified by two rounds of PCR. The first-round PCR was performed using primers 35S-F/35S-TMV R and TMV 5’ F/TMV 5’ R to generate DNA fragments containing 35S promoter from pBI121 and part of TMV, which is upstream of *Xho*I from p30B, respectively. The two DNA fragments were subsequently fused together by PCR with primers 35S-F/TMV 5′R. The DNA fragments were inserted into pGEM-T Easy (Promega, Madison, WI, USA, Cat. No. A1360). A ribozyme [97] was added at 3′ end of *tobacco mild green mosaic virus* (TMGMV) in p30B-GFPC3 by two rounds of PCR with primers 30B 3’-F/30B 3’-R1 and 30B 3’-F/30B 3’-R2. The fragment with ribozyme was inserted into the pGEM-T Easy vector with 35S-TMV5′ that was in positive orientation between *Xho*I and *Sac*I sites. The DNA fragment was inserted into pBI121 between *Hin*dIII and *Sac*I sites to get the intermediate vector. Later, the putative virulence factors without signal peptides were amplified from HLB-infected citrus genomic DNA and inserted into p30B-GFPC3 between *Pac*I and *Xho*I sites. The plasmid containing the putative virulence factor was digested with *Xma*I and *Xho*I, and the released fragment harboring the putative virulence factor was isolated and ligated with the intermediate vector, to get the final plasmid for agroinfiltration.

#### 4.3.2. Construction of Plasmids for Subcellular Localization

For subcellular localization of the virulence factors that showed strong symptoms in *N. benthamiana*, pBI121 was used. Green fluorescent protein (GFP) was used for subcellular localization assay. The virulence factors were amplified by PCR to introduce *Bam*HI and *Xm*aI at 5′ and 3′ ends of the amplified fragments, respectively. Similarly, *Xma*I and *Sac*I sites were introduced at 5′ and 3′ of GFP fragment by PCR, and the purified DNA fragment was cloned into pGEM-T Easy vector. GFP fragment digested with *Xma*I and *Sac*I was inserted into the plasmid with virulence factor that was digested with the same enzymes, to get the plasmid with virulence factor-GFP DNA fragment. The GFP-targeted virulence-factor DNA fragment was cut with *Bam*HI and *Sac*I, and then it was inserted into pBI121, to obtain the plasmid for subcellular localization study.

#### 4.3.3. Construction Plasmids for Yeast Two-Hybrid Assay

Vector pGBKT7 DNA-BD (Clontech, Mountain View, CA, USA, Cat. No. 630443) was used to express the identified virulence factors for yeast two-hybrid. The virulence factors without signal peptide were inserted into pGBKT7 DNA-BD, between *Eco*RI and *Bam*HI sites. All the restriction enzymes were purchased from NEB (Ipswich, MA, USA).

### 4.4. Agroinfiltration

The six true-leaf stage plants were used for agroinfiltration. Plasmids were introduced into *Agrobacterium tumefaciens* strain EHA 105 [101] by electroporation. Agrobacterium cultures were adjusted at optical density (OD) 0.6 with solution (10 mM MgCl_2_, 10 mM MES pH 5.6, 10 µM acetosyringone) [98], and the first two true leaves were infiltrated with a 1 mL needleless syringe. At least three replications with 6 plants per replication were performed for each candidate gene.

### 4.5. Protein Extraction and Immunoblotting

*N. benthamiana* leaves were ground into fine powder with liquid nitrogen, for protein extraction. The extraction buffer contained 50 mM of sodium phosphate pH 7.0, 200 mM of NaCl, 10 mM of MgCl_2_, 10% glycerol, and one mini cocktail tablet (Roche, Basel, Switzerland)/10 mL solution [99]. Approximately 100 mg of fine powder was transferred into 100 µL extraction buffer, mixed, and centrifuged at 13,000 × *g*, for 30 min at 4 °C. The supernatant was used for immunoblotting. Proteins were separated on a 10% polyacrylamide gel and transferred onto polyvinylidene fluoride (PVDF) membrane with Trans-Blot^®^ Turbo™ Transfer System (Bio-Rad, Hercules, CA, USA). The membrane was stained with Ponceau S, followed by a rinsing with distilled water. The red bands on the membrane served as protein loading controls. The membrane was blocked with 4% BSA in TBS, for two hours, at room temperature, and incubated with anti-HA antibody H9658 (Sigma-Aldrich, St. Louis, MO, USA), overnight, at 4 °C, followed by the washing of the membrane with TBS three times (each time for 5 min). The blocked membrane was incubated with AP-labelled secondary antibody S3721 (Promega) for one hour, followed by a thorough washing. The signal was detected with Western Blue^®^ Stabilized Substrate for Alkaline Phosphatase (Promega, Cat. No. S3841).

### 4.6. Determination of the Subcellular Localization of Identified Virulence Factors

The six true leaf stage *N. benthamiana* was used for agroinfiltration to determine the subcellular localization of the putative virulence factors which caused strong phenotypes in *N. benthamiana*. The infiltrated leaves were harvested at 48 h after infiltration and observed under Olympus 152 FV1000 MPE multiphoton laser scanning microscope (Tokyo, Japan). When the distribution pattern of the fusion protein in plant cells was similar to free GFP, mRFP-VirD2NLS [42] was used to co-infiltrate with the GFP fusion protein to determine whether the fusion protein was located in the nucleus. Red fluorescent protein (RFP) targeting different organelle [41] was used to co-infiltrate with GFP fusion protein when the GFP fusion protein had different distribution patterns from free GFP.

### 4.7. Yeast Two-Hybrid

To probe the host proteins interacting with the putative virulence factors, yeast two-hybrid (Y2H) was carried out. RNAs from HLB-infected sweet orange ‘Valencia’ leaves were used for the construction of cDNA library [66]. The library construction was performed by following the manufacturer instructions for “Mate & Plate” Library System (Clontech, Cat no. 630490) with linearized pGADT7-Rec vector and yeast strain Y187. The Y2H assay was performed according to Matchmaker™ Gold Yeast Two-Hybrid (Clontech, Cat. No. 630489). Baits were cloned into vector pGBKT7 DNA-BD between *EcoR*I and *Bam*HI and transformed into yeast strain Y2H Gold. The autoactivation and toxicity of the baits were determined before screening the library. The annotations and accession numbers of positive prey were retrieved from national center for biotechnology information (NCBI, Bethesda, MD, USA). The orthologs of the prey in *Arabidopsis* were retrieved from the *Arabidopsis* information resource (TAIR) by BLASTP, with the default settings.

## Figures and Tables

**Table 1:** The selected putative virulence factors of *Ca*Las and the phenotypes of *N. benthamiana* expressing the putative virulence factors.

Locus Tag	Length	MW	SignalP	SignalP	Phobius	TMHMM	Annotation ^c^	Phenotype ^d^
	(aa) ^a^	(kDa) ^b^	4	3				
CLIBASIA_00070	409	47.62	N	Y	N	1	hypothetical protein	4
CLIBASIA_00100	209	23.91	Y	Y	Y	0	putative ABC transporter protein	2
CLIBASIA_00420	161	17.87	Y	Y	Y	1	outer membrane lipoprotein	2
CLIBASIA_00460	99	11.29	Y	Y	Y	0	hypothetical protein	2
CLIBASIA_00470	52	5.96	N	Y	Y	1	hypothetical protein	1
CLIBASIA_00525	98	11.56	N	Y	Y	0	hypothetical protein	2
CLIBASIA_00530	98	11.38	N	Y	Y	0	hypothetical protein	2
CLIBASIA_00965	288	31.76	Y	Y	Y	1	putative membrane-bound lytic murein transglycosylase signal peptide protein	2
CLIBASIA_01135	310	35.15	Y	Y	N	0	substrate-binding region of ABC-type glycine betaine transport system	4
CLIBASIA_01345	666	75.38	N	N	N	0	Serralysin, TISS	4
CLIBASIA_01555	321	36.61	N	N	N	0	hemolysin protein, TISS	4
CLIBASIA_01640	142	16.08	Y	Y	Y	1	hypothetical protein	2
CLIBASIA_02075	397	46.04	Y	Y	Y	0	chemotaxis protein	4
CLIBASIA_02120	295	33.18	Y	Y	Y	0	periplasmic solute binding protein	2
CLIBASIA_02145	211	23.59	Y	Y	Y	0	hypothetical protein	2
CLIBASIA_02180	272	31.41	N	N	N	1	Signal peptide protein	2
CLIBASIA_02215	121	13.91	N	Y	Y	1	hypothetical protein	2
CLIBASIA_02250	196	22.61	Y	Y	N	0	extracellular solute-binding protein	2
CLIBASIA_02275	71	8.25	N	N	Y	1	hypothetical protein	2
CLIBASIA_02305	193	21.92	N	N	Y	0	hypothetical protein	2
CLIBASIA_02425	206	21.63	Y	Y	Y	0	outer membrane protein	2
CLIBASIA_02470	131	14.77	Y	Y	Y	0	hypothetical protein	2
CLIBASIA_02845	200	23.41	N	Y	N	0	hypothetical protein	2
CLIBASIA_02935	490	53.54	N	Y	N	1	serine protease DO-like protease	4
CLIBASIA_03020	295	33.45	N	Y	Y	1	zinc uptake ABC transporter	2
CLIBASIA_03070	474	51.84	Y	Y	Y	0	putative pilus assembly protein	4
CLIBASIA_03085	121	12.87	N	Y	N	1	hypothetical protein	2
CLIBASIA_03120	61	7.02	Y	Y	Y	0	hypothetical protein	2
CLIBASIA_03230	162	17.76	Y	Y	Y	0	hypothetical protein	2
CLIBASIA_03515	347	39.38	N	N	N	2	uroporphyrinogen decarboxylase	4
CLIBASIA_03695	113	12.47	N	Y	Y	0	hypothetical protein	2
CLIBASIA_03915	41	4.51	N	Y	Y	1	hypothetical protein	2
CLIBASIA_03975	208	23.67	N	Y	N	1	hypothetical protein	2
CLIBASIA_04025	97	11.15	N	Y	Y	0	hypothetical protein	1
CLIBASIA_04030	89	9.37	N	Y	Y	0	hypothetical protein	2
CLIBASIA_04040	160	17.3	Y	Y	Y	0	hypothetical protein	3
CLIBASIA_04065	409	47.32	N	Y	N	1	hypothetical protein	C-terminal 1
CLIBASIA_04140	409	47.31	N	Y	N	1	hypothetical protein	4
CLIBASIA_04250	50	5.39	N	Y	Y	0	hypothetical protein	2
CLIBASIA_04320	216	24.74	N	Y	Y	0	hypothetical protein	2
CLIBASIA_04330	230	26.13	N	Y	Y	1	hypothetical protein	2
CLIBASIA_04405	122	13.65	N	N	Y	0	hypothetical protein	2
CLIBASIA_04410	123	14.34	N	Y	Y	0	hypothetical protein	2
CLIBASIA_04425	126	14.99	N	Y	Y	0	hypothetical protein	2
CLIBASIA_04470	280	30.61	N	N	N	8	hypothetical protein	2
CLIBASIA_04520	305	35.45	Y	Y	Y	0	hypothetical protein	4
CLIBASIA_04530	86	9.24	N	Y	Y	0	hypothetical protein	2
CLIBASIA_04540	412	47.8	N	Y	N	1	hypothetical protein	4
CLIBASIA_04560	195	21.77	Y	Y	Y	0	hypothetical protein	2
CLIBASIA_04580	117	13.35	N	Y	Y	0	hypothetical protein	2
CLIBASIA_04735	163	18.68	N	Y	Y	0	outer membrane lipoprotein omp19	2
CLIBASIA_05050	399	46.62	N	N	Y	1	von Willebrand factor type A	4
CLIBASIA_05060	421	48.23	N	N	N	1	von Willebrand factor type A	4
CLIBASIA_05115	286	21.18	Y	Y	Y	0	hypothetical protein	2
CLIBASIA_05150	225	24.81	Y	Y	Y	0	hypothetical protein	1
CLIBASIA_05320	86	9.58	Y	Y	Y	0	hypothetical protein	2
CLIBASIA_05330	68	7.76	N	N	N	0	hypothetical protein	2
CLIBASIA_05480	253	29.08	N	Y	N	2	hypothetical protein	2
CLIBASIA_05570	81	8.29	N	Y	Y	1	hypothetical protein	2
CLIBASIA_05640	69	7.67	Y	Y	Y	0	hypothetical protein	3

aa ^a^: amino acid; KDa ^b^: molecular weight in kilo Daltons. Annotation ^c^ was derived from *Ca*Las genome sequence in NCBI. Phenotype ^d^: 1. Show severe symptoms; 2: similar to TMV-GFP control; 3: attenuate the TMV symptoms; 4. Symptomless in systemic leaves.

**Table 2:** The identified host proteins interacting with the four virulence factors by yeast two-hybrid.

Bait	Accession Number	Annotation	Isolate
CLIBASIA_00470	XM_006480567	polyubiquitin-like	1
	XM_006480570	polyubiquitin-like	1
	XM_006475150	polyubiquitin-like	1
	XM_006495169	agglutinin-2-like	6
	XM_006489869	galactinol--sucrose galactosyltransferase 2-like	13
	XM_006466177	DnaJ homolog 1 like	7
	XM_006470378	NDR1/HIN1-like protein 13	2
	XM_006470373	L-ascorbate oxidase-like	1
	XM_006473640	cinnamyl alcohol dehydrogenase 6-like	1
	XM_025093156	heat shock 70 kDa protein 6	1
	XM_006466284	8-hydroxygeraniol dehydrogenase-like	1
	XM_006486253	phosphoinositide phospholipase C 2-like	1
	XM_025092931	TSS protein	1
	XM_006475911	UPF0587 protein C1orf123	1
	XM_006473608	thaumatin-like protein 1b	1
	XM_006469523	uncharacterized protein	1
	XM_006479243	uncharacterized protein	1
CLIBASIA_04025	XM_006495169	agglutinin-2-like	4
	XM_006489869	galactinol--sucrose galactosyltransferase 2-like isoform	1
	XM_006465840	subtilisin-like protease-like	1
	XM_006470378	NDR1/HIN1-like protein 13	1
	XM_006485422	DnaJ protein homolog	1
	XM_006486253	phosphoinositide phospholipase C 2-like	1
	XM_006489870	probable galactinol--sucrose galactosyltransferase 2	1
	XM_006486194	cytosolic endo-beta-N-acetylglucosaminidase 1-like	1
	XM_006473608	thaumatin-like protein 1b	1
	XM_006472735	disulfide-isomerase	1
CLIBASIA_05150	XM_006474664	cysteine protease Cp	3
	XM_006471352	uncharacterized protein	1
	XM_025096339	ribose-5-phosphate isomerase 3	1
	XM_006441377	Kunitz trypsin inhibitor 2	1
	XM_006492080	dirigent protein 16	1
	XM_006485840	elongation factor 1-alpha	1
CLIBASIA_04065	orange1.1g037576m	hypothetical protein	1

## Supplementary Materials

Supplementary materials can be found at https://www.mdpi.com/1422-0067/20/22/5575/s1.

## Abbreviations

*Ca*Las	*Candidatus* Liberibacter asiaticus
TMV	tobacco mosaic virus
GFP	green fluorescent protein
RFP	red fluorescent protein
*N. benthamiana*	*Nicotiana benthamiana*
CTV	Citrus tristeza virus
Y2H	yeast two-hybrid
HLB	Huanglonbbing
HR	hypersensitive response
PCD	programmed cell death
SA	salicylic acid
ROS	reactive oxygen species
SP	signal peptide
CP	cysteine protease
