# Supplementary material for: Identification of the Virulence Factors of Candidatus Liberibacter asiaticus via Heterologous Expression in Nicotiana benthamiana using Tobacco Mosaic Virus
Source: Int J Mol Sci. 2019 Nov 8;20(22):5575. doi: 10.3390/ijms20225575 (PMC6888081; doi:10.3390/ijms20225575)
Supplement: Supplementary file 1 [file ijms-20-05575-s001.pdf]

International Journal of
Molecular Sciences

Supplementary Materials

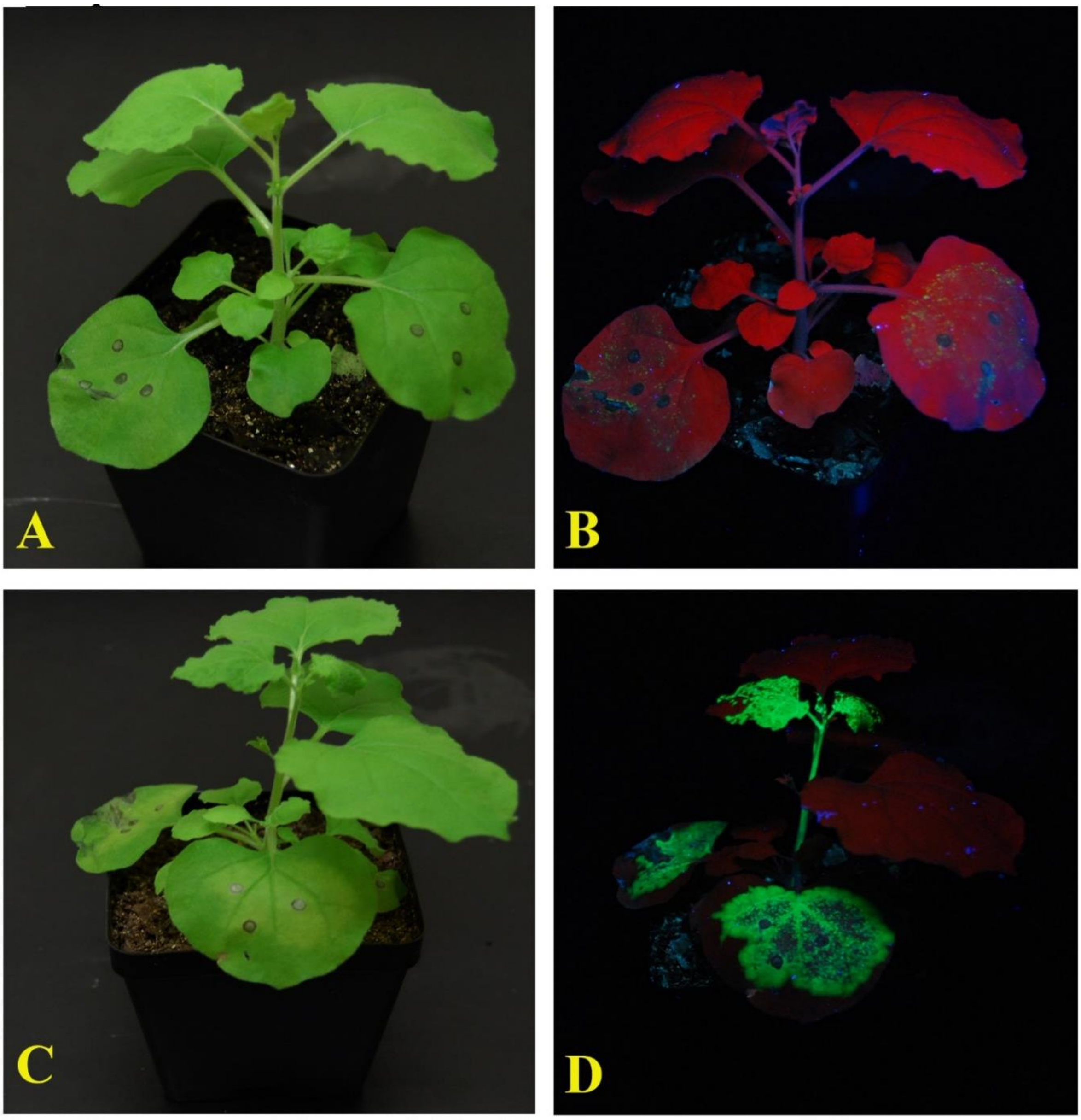

Supplementary Figure 1. TMV-GFPC3 could replicate in infiltrated *N. benthamiana* leaves and move to systemic leaves by agroinfiltration.

N. benthamiana was infiltrated with EHA105 harboring pBI121-30B-GFPC3. (**A**) and (**B**) were taken at 5 DPI, (**A**) under normal light and (**B**) under UV light. (**C**) and (**D**) were taken at 10 DPI, (**C**) under normal light and (**D**) under UV light. GFP was observed in the infiltrated leaves under UV light (**B**), and systemic leaves were green under UV light at 10 DPI (**D**).

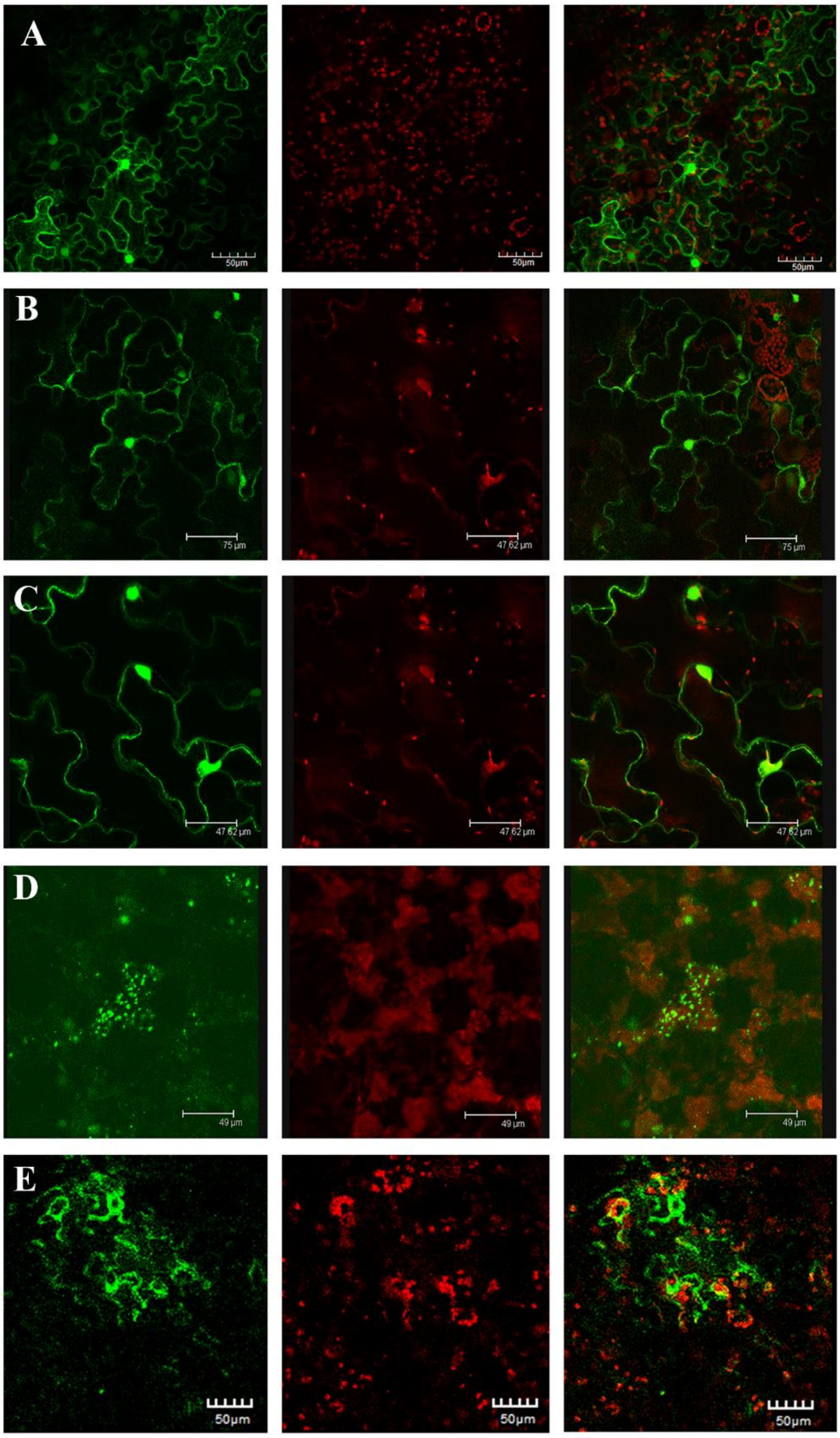

Supplementary Figure 2. Distribution patterns of free GFP, CLIBASIA_00470-GFP, CLIBASIA_04025-GFP, CLIBASIA_05150-GFP and CLIBASIA_04065-GFP in *N. benthamiana*. CLIBASIA_00470-GFP (**B**) and CLIBASIA_04025-GFP (**C**) distributed similarly in *N. benthamiana* to free GFP (**A**), but CLIBASIA_05150-GFP (**D**) and CLIBASIA_04065-GFP (**E**) distributed differently. Left row showed GFP, middle showed chloroplast, right showed overlay of GFP and chloroplast. Free GFP distribute in in the cytosol and to the nuclear lumen; CLIBASIA_05150-GFP showed small green dots; CLIBASIA_04065-GFP showed green dots, cycles, and grain-like shape.

International Journal of *Molecular Sciences*

MDPI

Supplementary Table 1. Primers used in this research.

**1. Primers for Screening the Putative Virulence Factors**	
**1A. Primers for Construction Intermediate Vector**	
35S-F	*AAGCTT*CCCCAGATTAGCCTTTTCAATTTC
35S-TMV R	GTTTGTTGTTGTTGGTAATTGTTGTAAAAATACTTCTCTCCAAATGAAATGAACTTC
TMV 5' F	GAAGTTCATTTCATTTGGAGAGAAGTATTTTTACAACAATTACCAACAACAACAAAC
TMV 5' R	*CTCGAG*GTAATTTGGAATTCTGGATACG
30B 3'-F	*CTCGAG*GGGTAGTCAAGATGCATAATAA
30B 3'-R1	CTTTAGGGACTCGTCAGTGTACTGATATAAGTACAGACTGGGCCGCTACCCGCGGTTAG
30B 3'-R2	*GAGCTC*TGCAGATATCCCAGTTTCGTCCTTTAGGGACTCGTCAGTGTACTGATATAAG
**1B. Primers for Expression the Selected Putative Virulence Factors with TMV via Agroinfiltration**	
CLIBASIA_00070 F	*TTAATTAATG*GATGTTGTGGTAGATCAGGTGACGGTG
CLIBASIA_00070 R	*CTCGAG*CTAGCTATCGACCACAGTAATTGTAAACTGAC
CLIBASIA_00100 F	*TTAATTAATG*AATCCTGTAAATATATACGATTTAAC
CLIBASIA_00100 R	*CTCGAG*CTAATTGTCACTTAAAGGAAGAGATG
CLIBASIA_00420 F	*TTAATTAATG*GCCATAGCTGTGGTTTCTTGTAGTGC
CLIBASIA_00420 R	*CTCGAG*CTATTTATAAAATATGATTGACGTAG
CLIBASIA_00460 F	*TTAATTAATG*CAAGTTTATCATATCCATTCGCCTC
CLIBASIA_00460 R	*CTCGAG*CTATTTTTTATCTTCTTCAAATAAATATTTG
CLIBASIA_00470 F	*TTAATTAAATG*GGTGGGCTATGCTTTAACCGCCCAAAAG
CLIBASIA_00470 MF	*TTAATTAATAA*GGTGGGCTATGCTTTAACCGCCCAAAAG
CLIBASIA_00470 R	*CTCGAG*TTATTGGTAGCGCCTTTCTCTTGCTTC
CLIBASIA_00525 F	*TTAATTAATG*TATGAAGATGCGATCAGGTCTCAATTTG
CLIBASIA_00525 R	*CTCGAG*TTACTCTTTCTCATTTTGCTCAATGG
CLIBASIA_00530 F	*TTAATTAATG*GATGACAGGATTACGGAATTAAATAC
CLIBASIA_00530 R	*CTCGAG*TTATTGTTCAAGGGTTGTGGTTTTAC
CLIBASIA_00965 F	*TTAATTAATG*GTAACGCAACCTAAGAAAGCAACAAC
CLIBASIA_00965 R	*CTCGAG*TTATGTATCGTATCCTTTTCCTATTG
CLIBASIA_01135 F	*TTAATTAATG*AGAGATGCCGATTCTTGTACGCC
CLIBASIA_01135 R	*CCTCGAG*TTAATTATTCATAAATCTCTCTAGTTG
CLIBASIA_01345 F	*ATTAATTAATG*CATAATATAAAACCGGTTTATAC

CLIBASIA_01345 R	*CCTCGAG*TCAGGAAAAATCATGATTTATATCATG
CLIBASIA_01555 F	*ATTAATTAATG*ATGAGCGATTTTAAAATAAATTCATC
CLIBASIA_01555 R	*CCTCGAG*TTAATTTTTAAAATGAACAAGACGTC
CLIBASIA_01640 F	*TTAATTAATG*GGTTTTCGGGTTTGTAATGGCAC
CLIBASIA_01640 R	*CTCGAG*TTATCTCCTATCTTGTGCAGGCTCAG
CLIBASIA_02075 F	*TTAATTAATG*ACGGATCAGGATTTGGTTCGTACTATTG
CLIBASIA_02075 R	*CTCGAG*TCATCTCGCTTCTGCAAGCAAAACATC
CLIBASIA_02120 F	*TTAATTAATG*ACGACACAAAAAAAAGTAGTATTATCCAG
CLIBASIA_02120 R	*CTCGAG*TCAAAATAAAGTATCCACAATTTTCG
CLIBASIA_02145 F	*TTAATTAATG*GCTAGATTCGCAAATAAAGTAGCCG
CLIBASIA_02145 R	*CTCGAG*CTATTTGCTGATCTGAACGTCATTATG
CLIBASIA_02180 F	*ATTAATTAATG*CGGTTAGAGAATGGTCTTGCAATATC
CLIBASIA_02180 R	*CCTCGAG*TTACATTTTTGATGGCACAAAAAAAAAGTG
CLIBASIA_02215 F	*TTAATTAATG*GAAGACAACAGGATAGAGAGTTTG
CLIBASIA_02215 R	*CCTCGAG*TTAGTAATCCGATACTTTCTCCCCA
CLIBASIA_02250 F	*TTAATTAATG*ACGGAAAATACTACCAAATATTTG
CLIBASIA_02250 R	*CCTCGAG*CTATCGCTTGTATTTGGTCAATATC
CLIBASIA_02275 F	*TTAATTAATG*ATTCAAATTTATTCTCCTATATCTG
CLIBASIA_02275 R	*CTCGAG*TTATTTGACAAAAGATCCCCTCAG
CLIBASIA_02305 F	*TTAATTAATG*GATCATTCAGATTCACAGCATCCTCCAG
CLIBASIA_02305 R	*CTCGAG*TTATTCTTCCATTTTCGGTGGATCCTG
CLIBASIA_02425 F	*TTAATTAATG*GCTGATCCTGTGCGTCGTGCTCATC
CLIBASIA_02425 R	*CTCGAG*TTAAAACTTCATTCCTACACCCAAAG
CLIBASIA_02470 F	*TTAATTAATG*CTTAATTGCAACGAAACTTTAATGCAAG
CLIBASIA_02470 R	*CTCGAG*TCAATTATTTATAAATGGGCAGAGCAG
CLIBASIA_02845 F	*TTAATTAATG*AATACAGCAAATATCTTAATCCCATC
CLIBASIA_02845 R	*CCTCGAG*TTACTGACAGTGTAGGCGTGG
CLIBASIA_02935 F	*TTAATTAATG*TTAGAGGCTAAGCTTCCCCCATCC
CLIBASIA_02935 R	*CTCGAG*TTATTTGTCAATTTTTAGAGAAACG
CLIBASIA_03020 F	*TTAATTAATG*ATACTTATATTTCTTTTTTTTATATTG
CLIBASIA_03020 R	*CTCGAG*CTAAGAACAGTTCTTAGCTATTGAAT
CLIBASIA_03070 F	*TTAATTAATG*AAGTTACCACCAATTAAAGAGGCAAATGC

CLIBASIA_03070 R	*CTCGAG*TCATTTATAAATAAAACCAATTGCACC
CLIBASIA_03085 F	*TTAATTAATG*GATAGGATGAAAGGTGTTTACCAG
CLIBASIA_03085 R	*CTCGAG*TCACGAGCTTTTATTTGGCTTAAC
CLIBASIA_03120 F	*TTAATTAATG*CAAGATGAAATTAAGAAGAATAATCC
CLIBASIA_03120 R	*CTCGAG*TTACGATGCAAAGGAGGTAAAGGCGGC
CLIBASIA_03230 F	*TTAATTAATG*CTTCTTACGAAAAAGATTGAAAGTG
CLIBASIA_03230 R	*CTCGAG*TCAGGGACATAAACCCTTTGACGTCG
CLIBASIA_03515 F	*TTAATTAATG*AATGAACGAAAACAAAAGATTCTTG
CLIBASIA_03515 R	*CTCGAG*TTAAATCTTCTCCGAACGTACAGTT
CLIBASIA_03695 F	*TTAATTAATG*TTTGAAAAATATAAAGCGCC
CLIBASIA_03695 R	*CTCGAG*TCAGAAGCTATAATATTGGGTA
CLIBASIA_03915 F	*TTAATTAATG*TGGAATCTAAAGCACGCAATTAGAAAG
CLIBASIA_03915 R	*CTCGAG*TTATTGTTCCTTTATTGCTATTTC
CLIBASIA_03975 F	*TTAATTAATG*CAAAATTTTCATGCAGTAGTGCC
CLIBASIA_03975 R	*CTCGAG*TTATGTCGCATTCATTGGCTGTTC
CLIBASIA_04025 F	*TTAATTAAATG*GATACTCTCTCTGACTCTAAGCAAC
CLIBASIA_04025 MF	*TTAATTAATAA*GATACTCTCTCTGACTCTAAGCAAC
CLIBASIA_04025 R	*CTCGAG*TTATCTTTCCCATTCTCTAACGTATCTTTC
CLIBASIA_04030 F	*TTAATTAATG*TCTGACCCCGCTAAAAAGAGTAACATC
CLIBASIA_04030 R	*CTCGAG*TTAGGCTCCTTCTTTTTTATCCTCTTTTG
CLIBASIA_04040 F	*TTAATTAATG*GATCCTGTAGCTCCACCACCACC
CLIBASIA_04040 R	*CTCGAG*CTAATCCTCTTTTGTGTTTTCTGC
CLIBASIA_04065 CF	*TTAATTAATG*TCACAAATTCCTTATAAAAATTTC
CLIBASIA_04065 R	*CTCGAG*CTAGCTATCGACCACAGTAATTGTAAAC
CLIBASIA_04065-HA R	*CTCGAGCTAAGCGTAATCTGGAACATCGTATGGGTA*GCTATCGACCACAGTAATTG
CLIBASIA_04065 CFSF	*TTAATTAATGCC*TCACAAATTCCTTATAAAAATTTC
CLIBASIA_04065 FSR	*CTCGAGCTACAGCGTAATCTGGAACATCGTATGGGTA*GCTATCGACCACAGTAATTG
CLIBASIA_04140 F	*TTAATTAATG*GATGTTGTGGTAGATCAGGTGAC
CLIBASIA_04140 R	*CTCGAG*CTAGCTATCGACCACAGTAATTGTAAACTGAC
CLIBASIA_04250 F	*TTAATTAATG*TGTTCTTCTAAAAAAGGAGGTG
CLIBASIA_04250 R	*CTCGAG*CTATCTTCTGGATTGATTTTTACCAT
CLIBASIA_04320 F	*TTAATTAATG*CATGAGGATGAATTAAAGGTC

CLIBASIA_04320 R	*CTCGAG*TTAGAATTTATAAACATAATCAAGAC
CLIBASIA_04330 F	*TTAATTAATG*ACTCCACAAAACCATCTTCTCTTTTTTC
CLIBASIA_04330 R	*CTCGAG*TTATATAAAGAAGTGCATAGGATATTTTGC
CLIBASIA_04405 F	*TTAATTAATG*GACATTGTCATTGGTAGAACAGAGG
CLIBASIA_04405 R	*CTCGAG*CTAATTTGATGGGGGCACAGTGATTTTTTTAAC
CLIBASIA_04410 F	*TTAATTAATG*CCGTGCGGCATAGAGGAAGATAAC
CLIBASIA_04410 R	*CTCGAG*TTAGTAGCGGTGTCCGTTGTTTTGTG
CLIBASIA_04425 F	*TTAATTAATG*TATGATGCGAAAGCAAGAAGATTC
CLIBASIA_04425 R	*CTCGAG*TTACTGGGATCGGTAGTTTCGATAATATC
CLIBASIA_04470 F	*TTAATTAATG*TACGATTTATTATATAATTTTTC
CLIBASIA_04470 R	*CTCGAG*TTACGCAAAAACAAAGGATGTGG
CLIBASIA_04520 F	*TTAATTAATG*CAAAAAGTTAGACTTGTGTCGTGG
CLIBASIA_04520 R	*CTCGAG*TCAAAAATCATAATCGATGCTGATAG
CLIBASIA_04530 F	*TTAATTAATG*TTAGATCCGGAGAATGGAATAAG
CLIBASIA_04530 R	*CTCGAG*TTATTTTCTTAAAAAATTTTGTGATCTAAG
CLIBASIA_04540 F	*TTAATTAATG*GATGTTGTGGTAGATCAGGTGACGGTG
CLIBASIA_04540 R	*CTCGAG*TTACCGCCTCCAGCTATCGACCACAGTAATTG
CLIBASIA_04560 F	*TTAATTAATG*GATTCCATTGCAAAAAAAAGAAATAC
CLIBASIA_04560 R	*CTCGAG*TTAAAATGCTACGTCCCATAGCTTTAAC
CLIBASIA_04580 F	*TTAATTAATG*CAACCTTTTTTGGAAGAGACGG
CLIBASIA_04580 R	*CTCGAG*CTAATGATGCGACGGCAAAGGAGG
CLIBASIA_04735 F	*TTAATTAATG*TGTACACAAATAGATTTCGGAAAT
CLIBASIA_04735 R	*CTCGAG*CTACCGAGAAATTATAACTTTATCACTTTC
CLIBASIA_05050 F	*TTAATTAATG*ATCGATTTAGCGCACATTATGTAC
CLIBASIA_05050 R	*CTCGAG*TTATCTATTAGGTGCAATTCTCACAC
CLIBASIA_05060 F	*TTAATTAATG*CATTTATTAAGTAGATTCCG
CLIBASIA_05060 R	*CTCGAG*TTATCCTTTGAGGATTACACTATATTTAC
CLIBASIA_05115 F	*TTAATTAATG*TCACAACCTGAGCCTACATTACG
CLIBASIA_05115 R	*CTCGAG*TTAATTACATTTACGAATATCTGCC
CLIBASIA_05150 F	*TTAATTAAATG*GACTATGGGTATTCTCCCCAGTTTC
CLIBASIA_05150 MF	*TTAATTAATAA*GACTATGGGTATTCTCCCCAGTTTC
HA-CLIBASIA_05150 F	*TTAATTAATGTACCCATACGATGTTCCAGATTACGCT*GACTATGGGTATTCTCCCCAG

CLIBASIA_05150 R	*CTCGAG*TTAAAAGCGTAAAACCACACCAGCTGTG
CLIBASIA_05320 F	*TTAATTAATG*GCCAATGAGCACTCTTCTGTATC
CLIBASIA_05320 R	*CTCGAG*TCAATTGTTTAAGCCTCCAAAGAGC
CLIBASIA_05330 F	*TTAATTAATG*CTTCCACAAATTTTCTCTCTTATTAT
CLIBASIA_05330 R	*CTCGAG*TCAATATGTTGCAAAAAAAGAATCAAG
CLIBASIA_05480 F	*TTAATTAATG*GCGGCTCTGGCAACTCCTATAGG
CLIBASIA_05480 R	*CTCGAG*TTATCTTTGGGATCTTCGATTAAAAGC
CLIBASIA_05570 F	*TTAATTAATG*GCGGCTCTGGCAACTCCTATAGG
CLIBASIA_05570 R	*CTCGAG*TCAGTAGTTTTCTGTCTCTTCTTTAATC
CLIBASIA_05640 F	*TTAATTAATG*GATGAACCAAAGAAGCTGAATC
CLIBASIA_05640 R	*CTCGAG*TTAATCACTAGATAGTTTCGCACCCT
**2. Primers for Subcellular Localization**	
GFP F	*TCTAGA*ATGGCTAGCAAAGGAGAAGAACTTTTC
GFP R	*GAGCTC*TTATTTGTAAAGTTCATCCATGCCATGTGT
GFP FF	*CCCGGG*GCTAGCAAAGGAGAAGAACTTTTC
GFP FR	*GAGCTC*TTATTTGTAAAGTTCATCCATGCCATGTGT
CLIBASIA_00470 FF	*TCTAGA*TGGGTGGGCTATGCTTTAACCG
CLIBASIA_00470 FR	*CCCGGG*TTGGTAGCGCCTTTCTCTTGCTTC
CLIBASIA_04025 FF	*TCTAGA*TGGATACTCTCTCTGACTCTAAGC
CLIBASIA_04025 FR	*CCCGGG*TCTTTCCCATTCTCTAACGTATCTTTC
CLIBASIA_04065 FF	*TATAGA*TGGATGTTGTGGTAGATCAGGTG
CLIBASIA_04065 FR	*CCCGGG*GCTATCGACCACAGTAATTGTAAAC
CLIBASIA_05150 FF	*TCTAGA*TGGACTATGGGTATTCTCCCCAGTTTC
CLIBASIA_05150 FR	*CCCGGG*AAAGCGTAAAACCACACCAGCTGTG
RFP-NLS F	*GGATCC*ATGGCCTCCTCCGAGGACGTCATC
RFP-NLS R	*GAGCTC*TCTAGATCAGGTCGATCCCGACGCTATTTCCGAAG
**3. Primers for Yeast Two-Hybrid**	
CLIBASIA_00470 F	*GAATTC*ATGCCGTCTAAAGAAGAACAAGCACG
CLIBASIA_00470 R	*GCAGGTCGACGGATCC*TTATTGGTAGCGCCTTTCTCTTGC
CLIBASIA_04025 F	*CATGGAGGCCGAATTC*GATACTCTCTCTGACTCTAAGCAAC
CLIBASIA_04025 R	*GGATCC*TTATATATTGTTCTTTATCTTTATGGATG
CLIBASIA_04065 F	*GGCCGAATTC*GATGTTGTGGTAGATCAGGTGACGGTG

CLIBASIA_04065 R	*CGACGGATCC*CTAGCTATCGACCACAGTAATTGTAAACTG
CLIBASIA_05150 F	*CATGGAGGCCGAATTC*GACTATGGGTATTCTCCCCAGTTTC
CLIBASIA_05150 R	*GCAGGTCGACGGATCC*TTAAAAGCGTAAAACCACACCAGC

F for forward; R for reverse; MF for mutation forward; CFSF for C-terminal part frameshifting forward; FSR for frameshifting reverse; FF for fusion forward; FR for fusion reverse. Italic letters were for added sequences, underline for enzyme site.

Supplementary table 2. The height of *N. benthamiana* expression of *CLIBASIA_00470, CLIBASIA_04025, CLIBASIA_00470N, CLIBASIA_04025N* and *green fluorescent protein (GFPC3)* at 5 dpi and 15dpi.

**5 dpi**					**15 dpi**				
CLIBASIA_00470	*CLIBASIA_00470N*	*CLIBASIA_04025*	*CLIBASIA_04025N*	*GFPC3*	*CLIBASIA_00470*	*CLIBASIA_00470N*	*CLIBASIA_04025*	*CLIBASIA_04025N*	*GFPC3*
5	4.5	5	5	5	6.5	10.5	10	14	15
4.8	5	5.5	5.5	5	10	12.5	9.5	15	17
4.5	4.6	5.5	5.5	6	10	17	9.5	17	16
5	5	5	5.5	5	11.3	15	13	19	16
5.5	4.7	4.5	4.5	5	10.3	15	12	15	16
4.5	4.5	5	5	5	8.6	11.5	10	16	16

The height of plant measured in centimeter (cm). Six plants were used for each vector expressing *CLIBASIA_00470*, *CLIBASIA_04025*, *CLIBASIA_00470N*, *CLIBASIA_04025N* and control *GFPC3* via TMV. At 5 DPI, *N. benthamiana* infiltrated with different constructs were at similar height; *N. benthamiana* infiltrated with 30B-GFPC3, 30B-CLIBASIA_04025N, and 30B-CLIBASIA_00470N were at similar height and were much higher than plant infiltrated with 30B-CLIBASIA_00470 and 30B-CLIBASIA_04025 at 15 DPI. CLIBASIA_00470N: convert start codon of CLIBASIA_00470 to stop codon (TGA); CLIBASIA_04025N: convert start codon of CLIBASIA_04025 to stop codon TGA.
